# Draft genome sequences of the strains isolated from estuary sediments

**DOI:** 10.1128/MRA.00501-23

**Published:** 2023-11-10

**Authors:** Rameshkumar Dhanakoti, Vijayanand Dhanakoti, Karthik Loganathan, Mohandass Swathanthiram

**Affiliations:** 1 Research and Development, Salem Microbes Private Limited, Salem, Tamil Nadu, India; 2 Department of Biochemistry, PSG College of Arts and Science, Coimbatore, Tamil Nadu, India; Montana State University, Bozeman, Montana, USA

**Keywords:** probiotics, *Bacillus*, nanopore

## Abstract

Draft genome sequences of four strains of the *Bacillaceae* were generated using nanopore technology. The size of the genome ranges from 4.2 to 4.4 Mb. This study aims to enhance our understanding of the diversity of probable probiotic strains, their metabolic activities, and their safety in real-world applications.

## ANNOUNCEMENT

The family *Bacillaceae*, belonging to the phylum *Firmicutes* and class *Bacilli*, encompasses two important genera: *Bacillus* and *Clostridium*. The *Bacillus* genus comprises approximately 200 species and is widely distributed in nature. The members of this genus are commonly used as probiotics in various animal health industries impacting animal growth improvement, immunity enhancement, disease resistance, and water quality improvement of the rearing waters in aquaculture (1).

The strains utilized in this study were isolated in year 1999 from sediment samples collected from different parts of Tamil Nadu, India. These strains have been maintained in the SMPL (Salem Microbes Private Limited) microorganism collection. The isolation was done by serial dilution plating on tryptone (15.0 g), soya peptone (5.0 g), sodium chloride (25.0 g), agar (18.0 g), and tap water (1 L), pH 7.3–7.4. For DNA isolation, the strains (−80°C stored glycerol stock) were inoculated into Zobell marine broth 2216 (Himedia, India) at 28°C (120 rpm). After 24 hours of incubation, the pellets were separated using a centrifuge (9,000 rpm, 10 min), followed by washing with distilled water three times. Subsequently, DNA extraction was performed using the XpressDNA Bacteria Kit (MagGenome, India). The quantity and quality of DNA were analyzed using Qubit and agarose gel electrophoresis, respectively.

The sequencing library preparation was done using the SQK-LSK109 Kit (Oxford Nanopore Technologies, Oxford, UK) following the manufacturer’s instructions. The library was then loaded into a flow cell (R9.4 Chemistry), and a MinION sequencer was employed for DNA sequencing for 72 hours. The raw reads were base called using Guppy v6.1.5 in high accuracy mode. The reads were filtered using Filtlong v0.2.1, retaining 90% of the total reads with a minimum length of 1,000. Furthermore, the adapters were removed from the filtered reads using Porechop v0.2.4 (2). The reads were assembled using Flye v2.8 (3) and circularized using Circulator v1.5.5 (4). Finally, the assemblies were polished using Minimap2 v2.22 and Racon v1.4.3 (Table 1). The quality of the raw reads was verified using FastQC v0.11.9 (5). Subsequent annotation was carried out using RAST (6) and NCBI PGAP v6.3 (7). The Type (Strain) Genome Server (TYGS) was utilized to identify the genera and species (8).

**TABLE 1:** Strain details, sequence statistics, and data accession numbers of the strains used in this study

Microorganisms (TYGS)	*B. stercoris* SMPL704	*B. velezenis* strain SMPL710	*B. inaquosorum* strain SMPL713	*B. velezenis* SMPL715
Source	Sediment	Sediment	Estuarine mud	Sediment
Latitude and longitude	10.556347, 79.856060	10.1181072 N, 79.2270 E	10.404 N, 79.867 E	10.34785, 79.46715
Coverage	37	87	33	143
No. of reads	19423	114007	35238	121203
No. of contigs	2	2	3	2
N50 (bp)	41,82,320	41,59,213	41,84,062	41,93,954
Genome size (Mbp)	4.4	4.2	4.2	4.2
G+C content (%)	43.4	46.2	44.2	46.2
GenBank accession no.	JASMRE000000000	JAHRWM010000000	JAHRWN010000000	JASJOH000000000
SRA accession no.	SRR24744663	SRR15018744	SRR15018435	SRR24557201

Based on the TYGS analysis, strain 704 was assigned to the species *B. stercoris*, exhibiting a digital DNA-DNA hybridization (dDDH) (d4) value of 85.4% (95% CI, 82.7–87.8). The small difference in G+C content of 0.36% compared to the type strain *B. stercoris* D7XPN1 supports this assignment. Similarly, strains 710, 713, and 715 were assigned to the species *B. velezensis*, *B. inaquosorum*, and *B. velezensis*, respectively. The dDDH (d4) values for strains 710, 713, and 715 are 75.8% (95% CI, 72.8–78.6), 79.6% (95% CI, 76.7–82.3), and 75.8% (95% CI, 72.8–78.6), respectively. The slight G+C content differences of 0.13% (710), 0.38% (713), and 0.13% (715) compared to the type strains (*Bacillus velezensis* NRRL B-41580, *Bacillus inaquosorum* KCTC 13429, *Bacillus velezensis* NRRL B-41580, respectively) support these assignments.

## Data Availability

The whole genome sequences of these strains were submitted at DDBJ/ENA/GenBank under the accession numbers JASMRE000000000, JAHRWM010000000, JAHRWN010000000, and JASJOH000000000. The complete strains details are available in Table 1.

## References

[1] Soltani M, Ghosh K, Hoseinifar SH, Kumar V, Lymbery AJ, Roy S, Ringø E. 2019. Genus Bacillus, promising probiotics in aquaculture: aquatic animal origin, bio-active components, bioremediation and efficacy in fish and shellfish. Reviews in Fisheries Science & Aquaculture 27:331–379. doi:10.1080/23308249.2019.1597010

[2] Bonenfant Q, Noé L, Touzet H. 2023. Porechop_ABI: discovering unknown adapters in oxford nanopore technology sequencing reads for downstream trimming. Bioinform Adv 3:vbac085. doi:10.1093/bioadv/vbac085 36698762 PMC9869717

[3] Kolmogorov M, Yuan J, Lin Y, Pevzner PA. 2019. Assembly of long, error-prone reads using repeat graphs. Nat Biotechnol 37:540–546. doi:10.1038/s41587-019-0072-8 30936562

[4] Hunt M, Silva ND, Otto TD, Parkhill J, Keane JA, Harris SR. 2015. Circlator: automated circularization of genome assemblies using long sequencing reads. Genome Biol 16:294. doi:10.1186/s13059-015-0849-0 26714481 PMC4699355

[5] Wingett SW, Andrews S. 2018. FastQ screen: a tool for multi-genome mapping and quality control. F1000Res 7:1338. doi:10.12688/f1000research.15931.2 30254741 PMC6124377

[6] Aziz RK, Bartels D, Best AA, DeJongh M, Disz T, Edwards RA, Formsma K, Gerdes S, Glass EM, Kubal M, Meyer F, Olsen GJ, Olson R, Osterman AL, Overbeek RA, McNeil LK, Paarmann D, Paczian T, Parrello B, Pusch GD, Reich C, Stevens R, Vassieva O, Vonstein V, Wilke A, Zagnitko O. 2008. The RAST server: rapid annotations using subsystems technology. BMC Genomics 9:75. doi:10.1186/1471-2164-9-75 18261238 PMC2265698

[7] Tatusova T, DiCuccio M, Badretdin A, Chetvernin V, Nawrocki EP, Zaslavsky L, Lomsadze A, Pruitt KD, Borodovsky M, Ostell J. 2016. NCBI prokaryotic genome annotation pipeline. Nucleic Acids Res 44:6614–6624. doi:10.1093/nar/gkw569 27342282 PMC5001611

[8] Meier-Kolthoff JP, Göker M. 2019. TYGS is an automated high-throughput platform for state-of-the-art genome-based taxonomy. Nat Commun 10:2182. doi:10.1038/s41467-019-10210-3 31097708 PMC6522516

